# Control of Meningitis

**Published:** 1918-11

**Authors:** 


**Control of Meningitis.** By SIMON FLEXNER, *Journal of the American Medical Association.* August 24, 1918.

There may be differences of opinion as to just what part the ordinary healthy carrier of the meningococcus plays in initiating an outbreak of meningitis, but no one disputes the fact that without carriers of meningococcus, either healthy or sick, there can be no spread of disease.

Meningococcus is, of course, the essential factor, but without doubt secondary factors play important parts. Among the latter would appear to be excessive fatigue and concurrent respiratory infections of slighter degree, such as colds.

The source of meningitis in the army and navy is, in the first instance, the civilian population.

If we concentrate attention on the meningococcus itself, two essential facts appear : first, that numbers of meningococci are thrown off with the nasopharyngeal secretion; and second, that the bacteriologic type or variety of the organism plays a large part in determining the result. Some types or varieties of meningococci are more pathogenic than others.

Studies of the meningococci among the general civilian population and military personnel show that the healthy carriers tend to harbor not the highly parasitic, but rather the less pathogenic, varieties of meningococci. Once cases become frequent, however, the number of healthy carriers of parasitic kind rises.

We are permitted, therefore, to view the class of meningococci somewhat as we now view pneumococci. We distinguish among the latter parasitic and saprophytic varieties or types.

Should we proceed in the case of meningococcus as we have in that of pneumococcus, we should regard only the carriers of the parasitic varieties as menacing.

The distinction in type may be readily made on the basis of specific agglutination with monovalent immune serum derived from rabbits. The subject, as the author has outlined it, is in its early stages. It is to be hoped that the intrinsic importance of the matter will lead to a critical study of the meningococcus carrier problem, in the hope and expectation that the control of epidemic meningitis may be made simpler, more effective, and less disturbing to the training of our troops.

